# Gut microbiome sheds light on the development and treatment of abdominal aortic aneurysm

**DOI:** 10.3389/fcvm.2022.1063683

**Published:** 2022-11-25

**Authors:** Xuebin Ling, Wei Jie, Xue Qin, Shuya Zhang, Kaijia Shi, Tianfa Li, Junli Guo

**Affiliations:** ^1^Key Laboratory of Tropical Cardiovascular Diseases Research of Hainan Province, Department of Cardiovascular Medicine of the First Affiliated Hospital, Hainan Medical University, Haikou, China; ^2^Key Laboratory of Emergency and Trauma of Ministry of Education, Hainan Medical University, Haikou, China; ^3^Department of Medicine, Brigham and Women's Hospital and Harvard Medical School, Boston, MA, United States

**Keywords:** gut microbiome, abdominal aortic aneurysm, risk factors, pathogenesis, treatment

## Abstract

Abdominal aortic aneurysm (AAA) is an inflammatory vascular disease with high disability and mortality. Its susceptible risk factors include old age, being male, smoking, hypertension, and aortic atherosclerosis. With the improvement of screening techniques, AAA incidence and number of deaths caused by aneurysm rupture increase annually, attracting much clinical attention. Due to the lack of non-invasive treatment, early detection and development of novel treatment of AAA is an urgent clinical concern. The pathophysiology and progression of AAA are characterized by inflammatory destruction. The gut microbiota is an “invisible organ” that directly or indirectly affects the vascular wall inflammatory cell infiltration manifested with enhanced arterial wall gut microbiota and metabolites, which plays an important role in the formation and progression of AAA. As such, the gut microbiome may become an important risk factor for AAA. This review summarizes the direct and indirect effects of the gut microbiome on the pathogenesis of AAA and highlights the gut microbiome-mediated inflammatory responses and discoveries of relevant therapeutic targets that may help manage the development and rupture of AAA.

## Introduction

Abdominal aortic aneurysm (AAA) refers to the local full-thickness expansion of the subrenal aorta, in which the expansion diameter is >3 cm or >50% of the normal diameter. AAA is generally asymptomatic in the early stage, but the mortality rate can reach 80% following a rupture in the later stage (1). The main AAA risk factors are the age of >65 years, being male, and smoking history, while other risk factors include a family history of AAA, coronary heart disease(CHD), hypertension (HTN), peripheral artery disease, and previous myocardial infarction (2). The pathological characteristics of AAA mainly include an inflammatory response, vascular endothelial cells (VECs) damage, vascular smooth muscle cell (VSMC) apoptosis, extracellular matrix (ECM) degradation, and oxidative stress (3). Many leukocytes and inflammatory mediators are associated with the pathogenesis of AAA, including interleukin (IL)-1,−17, transforming growth factor (TGF)-β, and angiotensin II (AngII), which can infiltrate into aortic media and lead to smooth muscle cell depletion, generation of reactive oxygen species (ROS), and activation of matrix metalloproteinases (MMPs) causing ECM fragmentation (4).

The human gut microbiota is usually composed of four phyla: *Bacteroidetes* (9–42%), *Firmicutes* (30–52%), *Proteobacteria* (5–10%), and *Actinobacteria* (1–3%) (5), among which, *Bacteroides, Faecalis*, and *Bifidobacterium* are the most common genera in healthy adults. The pathogenic bacteria causing AAA originate from the skin, oral cavity, gastrointestinal tract, and respiratory tract and can infect the abdominal aorta through gut bacterial translocation, blood-borne transmission, and aortic aneurysm surgery. For example, *Streptococcus* colonizing the rectum can infect the abdominal aortic wall and induce AAA (6). *Propionibacterium acnes, Propionibacterium granulosum, Actinomyces viscosus, Actinomyces naeslundii*, and *Eggerthella lenta* are found in the aneurysm wall and intravascular plaque (7). Furthermore, the presence of *Campylobacter* and *Campylobacter urealyticus* may be related to the rupture of cerebral aneurysms (8). Additionally, compared to the non-AAA group, differences in gut microbiota were confirmed in both AAA mouse models (9) and patients with AAA (10). Gut microbiome dysbiosis can also cause oxidative stress injuries, as well as activate inflammatory cells and toll-like receptors (TLRs) through small molecules produced by microbial metabolites, which serve to intensify the remodeling of the arterial wall (11). Pro-inflammatory factors such as IL1-β, tumor necrosis factor (TNF)-α, interferon (IFN)-γ, and C-C motif chemokine ligand (CCL)-2 can accelerate the progression of AAA, while anti-inflammatory factors such as Arg1, IL10, and TGF-β can promote repair and prevent AAA rupture to a certain extent; therefore, the balance of these inflammatory factors regulated by the gut microbiome affects AAA pathological progression (12). These findings suggest that the gut microbiota contributes to the pathophysiology of AAA by modulating inflammation.

Nevertheless, the mechanism of the direct effect of gut microbiome species and metabolites on the pathogenesis of AAA is not so profound. The imbalance between symbiotic and pathological bacteria in the intestine may lead to changes in immune development and inappropriate inflammatory reactions, but it is unclear whether this imbalance is the cause or result of AAA. In this review, we focus on promoting the understanding of the pathogenesis of gut microbial-mediated AAA by summarizing the relevant findings of animal and human studies and seeking strategies to identify new therapeutic targets for AAA, with the aim to provide novel ideas for gut microbiome intervention in AAA.

## Association of AAA risk factors and the gut microbiome

### Atherosclerosis

Nearly 95% of patients with AAA have AS pathological changes, thus AS represents a vital independent risk factor for AAA (13). However, clinical anti-atherosclerotic drugs are incompletely effective for treating AAA, and AS plays a minor role in patients with AAA that are ≤ 45 mm (14). Recently, some investigations proposed that gut microbiome tightly linked AS with AAA. The changes at different stages of CHD were represented by *Roseburia, Klebsiella, Clostridium* IV, and *Ruminococcaceae*, which might affect AS by modulating the metabolic pathways of the host (15). A correlation between the diameter of AAA and gut microbiota of C57BL ApoE^(−/−)^ mice suggested that *Akkermansia, Odoribacter, Helicobacter*, and *Ruminococcus* play important roles in the progression of AAA (9). Furthermore, the AAA group had a higher relative abundance of *Leuconostocaceae, Ruminococcaceae, Weissella*, and *Faecalibacterium* and a lower relative abundance of *Firmicuteria, Selenomonadales*, and *Veillonellaceae* (10). Indeed, *Leuconostocaceae* is a risk factor for AAA and CHD, whereas an inverse association has been found between *Lachnospiraceae* and cardiovascular risk factors (16). Thus, the diversity of gut microbiome may be involved in the onset of AS and AAA.

### Aging

Although AAA is more common in males than in females, the prevalence increases with aging in both sexes (17). Increasing evidences show that the existence of aging cells greatly promotes the inflammatory state of aging blood vessels and can activate nuclear factor kappa-B(NF-κB), TLR, and MMPs in aging VECs, VSMCs, and ECM, resulting in an increased risk of AAA development (18, 19). The main characteristics of the gut microbiome composition of the elderly include reduced diversity, with *Bacteroidetes* as the dominant microbiota, and a lack of butyrate (20). Furthermore, a decrease in *Bifidobacteria* and increased levels of the mucin-degrading *Akkermansia muciniphila* have also been detected to a greater extent in elderly people compared to young adults (21). The decrease in probiotic-producing short-chain fatty acids (SCFAs) in the gut microbiome of the elderly plays a key role in the occurrence and progression of AAA. Aging can cause gut microbiome dysbiosis, leading to increased CD4^+^ T cell differentiation, and then trigger asystemic inflammatory response by bacterial-derived circulatory inflammatory factors. Aging also leads to oxidative stress by increasing TNF-α expression, which aggravates abnormal changes in the gut microbiota population of *Bifidobacterium* and the ratio of *Firmicutes*/*Bacteroidetes*, which leads to an increase in flavin-containing monooxygenase-3 and trimethylamine N-oxide (TMAO) (22).

### Smoking

Smoking is one of the most important modifiable risk factors of AAAs. Quitting smoking not only reduces the risk of developing an AAA but also limits the growth of AAA (23). It was demonstrated that smoking, age, and other factors can affect 8.87% of gut microbiome changes (24). Moreover, the relative abundance of *Prevotella* and *Neisseria spp*. is reduced for current smokers, and the relative abundance of *Firmicumis* increased, mainly *Streptococcus spp*., *Veillonella spp*., and *Rothia* (*Actinobacteria*) (25). Additionally, the differences in bacterial communities in current smokers may be related to the impairment of antibacterial defense caused by the immunosuppressive nature of tobacco (26). Indeed, it has been reported that nicotine from tobacco can stimulate the expression of MMPs in VSMCs, VECs, and inflammatory cells of the vascular wall and induce angiogenesis in aneurysm tissues (27).

### Obesity

Obesity is closely associated with the occurrence and progression of AAA (28). In human and mouse AAA lesions, IL18 co-localizes to its receptors at regions rich in adipocytes, which leads to AAA development through IL18 activation (29). Obesity can change the richness of microbes or their genes, and there is a stronger relationship between higher *Prevotella* relative abundance and body mass index in populations of different races (30). Obesity is also related to changes in the relative abundance of the two dominant bacterial divisions, the *Bacteroidetes* and the *Firmicutes* (31).

### Hypertension

AngII-induced HTN can increase the pressure of AAA, promote cardiac hypertrophy, damage VECs, and activate the inflammatory response of VSMCs, which are potential causes of the pathogenesis and progression of AAA (32). *Firmicutes/Bacteroidetes* ratio was increased and the acetate-, butyrate-, and lactic acid-producing bacterial populations were decreased in HTN rats and a patient model (33). *Lactococcus, Alistipes*, and *Subdoligranulum* abundances were positively correlated with systolic blood pressure or diastolic blood pressure in hypertensive patients (34). Furthermore, microbial richness and diversity were significantly reduced, gut probiotics were reduced, and other gut microbiome components such as *Prevotella* and *Klebsiella* were excessively increased in people with pre-HTN and HTN (35). Among them, *Klebsiella pneumoniae* was first reported to cause AAA (36). Evidence has also shown that the genus *Alistipes*, harbored in patients with HTN, is positively correlated with AAA diameter (37).

### Dyslipidemia

Low-density lipoprotein (LDL) cholesterol cause VECs damage, abnormal proliferation of VSMCs, and finally, AAA progression. The abundances of *Turicibacter, Lachnospira, Ruminococcus_gauvreauii*_group, and *Acetivibrio_ethanolgignens* _group increased in hyperlipidemic rats, while those of *Alistipes, Bacteroides, Ruminococcu*, and *Butyrivibrio* were decreased (38). A high-fat and high-fructose diet can alter the gut microbiota composition of Syrian hamsters, leading to dyslipidemia. Among these, *Ruminiclostridium* 9 and *Tyzzerella* were positively correlated with fasting cholesterol levels, while the *Tyzzerella* and *Ruminococceace* NK4A214 groups were positively correlated with fasting triglyceride levels (39). Moreover, the abundances of *Lachnospiraceae* and *Sutterellaceaecan* were significantly decreased, while that of *Prevotellaceae* was significantly increased in high-fat high-sugar-fed mice, and such a diet led to changes in metabolites and microbiota (40). An HFD not only leads to a reduction in antimicrobial peptides, but also increases inflammation and upregulation of CCL2, IL-β1, and MMPs (41). Besides, “Mediterranean” and vegetarian diets have anti-inflammatory effects and can increase gut probiotics except for improving dyslipidemia (42).

### Asthma

Asthma is an independent risk factor for AAA rupture (43). Low relative abundances of *Bifidobacterium, Ackermann, Lachnospira, Veillonella, Faecalibacterium, Rothia genera Ruminococcus gnavus*, and *Faecalis* and high abundances of *Candida, Rhodotorula* fungi, *Streptococcus* and *Bacteroides* were associated with the highest risk of developing childhood atopy and asthma (44–46). Our laboratory also reported that asthma-induced AAA development involves an inflammatory reaction through the activation of eosinophil-derived IL4, eosinophil cationic protein (ECP; cationic proteins of EOS), and IgE and mast cell activation (47).

### Chronic obstructive pulmonary disease

Chronic obstructive pulmonary disease (COPD) is independently and positively associated with AAA occurrence and rupture (48) but has no association with AAA growth (49). *Bifidobacteriaceae, Eubacteriaceae, Lactobacillaceae, Micrococcaceae, Streptococcaceae*, and *Veillonellaceae* were increased, whereas *Desulfovibrionaceae, Gastranaerophilaceae*, and *Selenomonadaceae* were decreased in patients with COPD (50). It was also found that the decrease in probiotics and the increase in *Enterobacteriacea*e and anaerobic bacteria can lead to an increased systemic inflammatory response, causing the occurrence and rupture of AAA (50).

### Chronic kidney disease

Chronic kidney disease (CKD) induces systemic inflammation, a condition considered to increase the risk of AAA incidence rate and abdominal aortic diameter enlargement (51). CKD severity is an important predictor of perioperative mortality and long-term survival after AAA repair (52). The bacterial families of *Actinobacteria, Firmicutes*, and *Proteobacteria* have been found to show the greatest increase in patients with CKD compared to healthy controls (53). Furthermore, *Bifidobacterium Catenulatum, Bifidobacterium longum, Bifidobacterium bifidum, Lactobacillus plantarum, Lactobacillus paracasei*, and *Klebsiella pneumonia* are known to be decreased in patients with peritoneal dialysis (54). The sharp reduction in gut probiotics reduces the ability of the gut microbiome to remove toxins, which can aggravate the systemic inflammatory response. Simultaneously, colon-derived uremic toxins can lead to gut microbiota imbalance and aortic wall damage, which can cause the onset and progression of AAA.

### Periodontitis

Periodontitis is highly prevalent in patients with both stable and unstable AAA, and *Porphyromonas gingivalis (Pg)* has been shown to be closely correlated with AAA diameters and volumes (55). In addition to *Pg, Aggregatibacter actinomycetemcomitans, Tannerellaforsythia*, and *Campylobacter rectus* are also involved periodontal pathogens in patients with AAA (56). *Pg* promotes AAA progression through systemic inflammation using the following mechanism: after entering the blood, *Pg* binds to the TLR-2 receptor of the abdominal aorta, induces overexpression of MMPs inside the AAA wall or thrombus, and enhances the intraluminal thrombus (ILT) enrichment (55, 57).

### Peptic ulcer disease

A previous study demonstrated that the incidence of peptic ulcer disease is 22.6% in patients with AAA compared to 7.2% in the general necropsy population (58). *Helicobacter pylori* infection can cause peptic ulcers, and one of the pathogenicity factors of *H. pylori* is the cytokine-associated gene A (CagA). Indeed, CagA^+^ *H. pylorico*-culture with *Lactobacillus acidophilus* has been shown to induce cytokine patterns (e.g., IL-2, IL-4, IL-6, IL-10, and IFN-γ), contributing to the pathogenesis of AAA (59).

### Diabetes mellitus

Interestingly, DM can cause arteriosclerosis, but the incidence or growth rate of AAA is lower in patients with diabetes (60). The gut microbiota is involved in insulin resistance, which is related to promote the progression of DM and AAA diameter (61–63). Importantly, in patients with newly diagnosed DM, the level of *Lactobacillus* is significantly increased, whereas the levels of *Clostridium coccoides* and *Clostridium leptum* are significantly decreased (64). Patients with DM also decreased the abundance of some universal butyric acid-producing bacteria, increased opportunistic pathogens, and rich functions of other microorganisms, all of which can reduce sulfate and stimulate bacterial defense mechanisms against oxidative stress injury (61, 65). Additionally, some hypoglycemic drugs, such as metformin, have the positive effect of increasing the life span of the gut probiotics (66), thereby making AAA less likely to occur or develop.

### Drugs

The existence of bidirectional interactions between microorganisms and drugs can be established through pharmacomicrobiomics (67), thus affecting the pathogenesis and progress of AAA. Doxycycline exposure results in *Bacteroides* expansion and *Bifidobacterium* and *Lactobacillus* reduction, which has an adverse impact on promoting AAA growth (68). Fluoroquinolones may also promote the occurrence or rupture of AAA (69). Moxifloxacin can reduce the abundance of *alistipes, bilophila, butyciromonas, coprobacillus, fecalibacter, odoribacter, oscillibacter, parasutterella, Roseburia*, and *sutterella* (70). Asthma medication can accelerate the growth or rupture of AAA. For instance, a combination of inhaled corticosteroids (ICSs) and oral glucocorticoids correlates positively with increased abundance of *Proteobacteria* and *Pseudomonas* and with decreased abundance of *Bacteroidetes, Fusobacteria*, and *Prevotella* (71). Patients with neutrophilic asthma using high doses of ICSs have been shown to have relative enrichment in *Haemophilus* and *Moraxella* species, members of the *Proteobacteria phylum*, and a reduced relative abundance of *Streptococcus, Gemella*, and *Porphyromonas taxa* compared to patients with eosinophilic asthma (72, 73). Additionally, other drugs (e.g., proton pump inhibitors, statins, metformin, β receptor blockers, ACE inhibitors, selective serotonin reuptake inhibitors, and antidepressants) are known to dramatically shift the microbiota profile and lead to less diverse changes in microbial composition (67).

**Figure 1:**
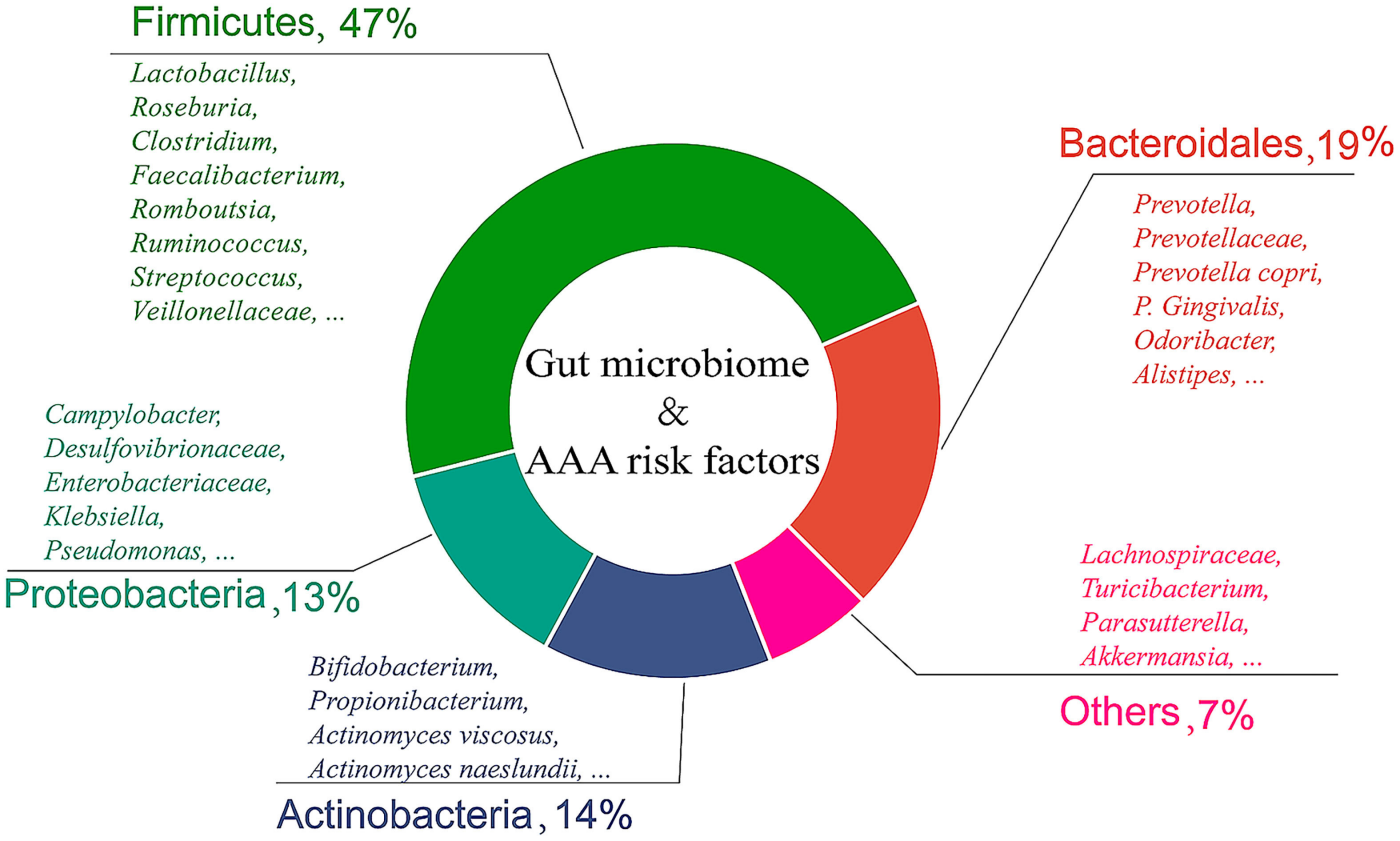
Composition ratio of gut microbiome related to AAA risk factors. This review lists 150 gut bacteria related to AAA, which are divided into five categories according to bacterial phyla.

Taken together, a total of five categories including 150 species of gut bacteria were related to AAA. The composition ratio of gut microbiome related to AAA risk factors is shown in Figure 1. Furthermore, the correlation between the gut microbiome and risk factors, and the pathological status of AAA are summarized in Supplementary Table 1.

## Immune cell-mediated pathogenesis of AAA *via* gut-aortic axis

### Lymphocytes

In the heart, aorta, and peripheral arteries, there is an increased accumulation of immune cells, including lymphocytes helper T cells (Th)1, Th2, Th17, and Tregs (74). IL-1, IL-6, IL-12, and TNF-α can lead to the accumulation of CD4^+^ T cells, whose effector cells can produce MMPs and cathepsins, causing aneurysm formation and rupture through damage to the aortic wall, inflammation, and loss of VSMCs (4). The systemic spread of inflammation is caused by a compromised gut barrier, and loss of antigen tolerance stimulates the differentiation of helper T cells to produce proinflammatory cytokines, including TNF-α, IL-1β, IL-6, IL-12, IL-23, and chemokines (75). *H. pylori* can also activate Th1 and Th2, causing AAA progression (59). Additionally, the role of *L. acidophilus* in inducing miRNA and apoptosis in CD4^+^ memory T cells was investigated and shown to directly affect the occurrence and prevention of AAA (76). *Bacteroides fragilis* polysaccharide A (PSA) has been shown to impact the Th1/Th2 ratio, which contributes to the development of the immune system. PSA is a component of the cell wall of *Bifidobacterium fragilis*, which induces the production of IL-10 through gut T cells (77), and the induction of Tregs has been shown to depend on IL-10-producing B cells. Segmented filamentous bacteria can promote the differentiation of Th17 cells (78), and activated Th17 cells play an important role in the occurrence and development of AAA. The interaction of gut bacteria with food mediates immune cell activation, cytokine production, and T lymphocyte proliferation through SCFA metabolism. Importantly, 80% of activated B cells in adults exist in the gut mucosal tissue. IgE action contributes to AngII perfusion-induced mouse AAA growth by increasing inflammation (79). In the process of AAA formation, immunoglobulin G (IgG) immune complexes infiltration interacts with activated Fc receptors (FcγR) in VSMCs to play a pathogenic role. Targeting FcγRs and/or downstream molecules to inhibit humoral immune damage in patients with AAA has become a new immunotherapeutic strategy (80).

### Neutrophils

Neutrophils are important inflammatory cells for AAA formation and progression, which are rich in MMPs and degrade ECM components. Neutrophils promote the transcription of IL-6 and pro-IL-1β in macrophages, inducing Th17 cell differentiation and recruiting more inflammatory cells (81). However, IL-17, which is produced by the Th17 cell, and the lack of IL-17 receptor may increase *Proteobacteria* and *Bacteroidetes phyla* and reduce *A. muciniphila*, which lead to increased commensal bacterial translocation into the bloodstream (82). Another study showed a significant increase in plaques in LDLR^(−/−)^ mice, together with increased neutrophil infiltration in the aortic root, and decreased concentrations of the anti-inflammatory SCFAs propionate, acetate, and butyrate in the cecum, suggesting that gut microbes can influence the level of neutrophils in the circulation and plaques to mediate plaque growth (83). Moreover, *Pg* is known to be involved in the pathogenesis of human AAA through neutrophil activation that is associated with neutrophil extracellular trap (NET) formation in the intraluminal thrombus (57). Neutrophil elastase and TNF-α levels were significantly elevated in aneurysm walls covered by thick layers of ILT, while neutrophil gelatinase-associated lipocalin, myeloperoxidase, and neutrophil elastase were positively correlated, and their mediators may infiltrate the thick AAA compartment and weaken the AAA wall (84).

### Macrophages

Aortic resident macrophages, blood-derived monocytes, and inflammatory macrophages are significantly expanded in the elastase-induced model of AAA (85). Initiate IL-6 production to accumulate monocytes/macrophages, activate STAT3 and monocyte chemoattractant protein-1 (MCP-1), and ultimately promote the dilation of AAA. Macrophages secrete MMPs to promote the degradation of vascular wall structural fibrin and secrete various pro-inflammatory factors to accelerate migration. Moreover, M1 macrophages can be activated by stimuli such as LPS and IFN-γ, aggravating local inflammation and promoting aortic dilation and vascular remodeling (86). A previous study introduced a Notch receptor inhibitor that upregulates M2 macrophages and downregulates M1 macrophages into ApoE^(−/−)^ mice with AAA, and the results showed significantly improved AAA progression (87). Gut microbiota also contributes to AAA development by regulating macrophage infiltration and inflammatory cytokine expression. Indeed, gut microbiome-dependent metabolites tryptophan and indole-3-acetate can inhibit fatty acids and LPS-stimulated secretion of pro-inflammatory cytokines in macrophages (88). Data have shown that antibiotic administration increases the gut microbiota *Firmicutes/Bacteroidetes* ratio and expression of CD68^+^ foam cells, with enhanced M1 polarization in plaques, thereby delaying inflammation within atherosclerotic plaque regression (89). More direct evidence suggests that *B. fragilis* supplementation in mice on an HFD reduces the abundance of *Lactobacillus* and increases the abundance of *Desulfovibrio*, resulting in increased macrophage accumulation in the small intestine and aortic tissue (90).

### Eosinophils

Shi Laboratory reported the occurrence and accumulation of EOS in AAA lesions, and the lack of EOS-aggravated AAA growth suggested that EOS plays a protective role in AAA (91). ECP has been shown to significantly alter the gut microbiota structure and promote the growth of probiotics in C57BL/6J mice (92). The same study also showed that ECP had different effects on male and female microbiota. In females, ECP increased the abundance of *Bifidobacterium* and *Akkermansia muscaria*, while in males, ECP increased the abundance of *Lactobacillus spp*. (92). Moreover, serum LPS-binding protein of gram-negative bacteria, a well-established biomarker for studying gut antigenic load, was shown to be significantly reduced by ECP to maintain normal gut homeostasis (92). ECP is a novel, food-based, anti-inflammatory agent that alleviates UC by modulating gut dysbiosis (93). Additionally, EOS deficiency leads to altered gut microbiota composition, which in turn may affect EOS function (94) and contribute to gut immune homeostasis.

### Mast cells

A previous study showed that increased MC counts are observed in the outer media and adventitia of the patient's AAA tissues; MCs directly enhance the activity of MMP9 produced by monocytes and macrophages (95), suggesting that MCs play a critical role in the progression of AAA. Moreover, patients with AAA have elevated MC proteases, such as chymotrypsin and tryptase, which contribute to leukocyte adhesion and migration, VSMC apoptosis, foam cell formation, and expression of MMPs and cathepsins (96). Additionally, MCs release IL6, IFN-γ, and β-FGF upon activation, induce VSMCs and VECs to express tissue-destructive cathepsins, and promote angiogenesis, which plays harmful roles in AS and AAA (97, 98). Evidence shows that LPS from *Rhodobacter sphaeroides* leads to the activation of MCs, causing impairment of the intestinal barrier function (99). Despite few reports on the interaction between gut microbes and MCs in the cardiovascular field, the direct or indirect relationship between gut microbes and MCs has been found in inflammatory diseases, such as irritable bowel syndrome (99). It is speculated that the imbalance of the gut microbiome causes MC activation, which will promote the progress of AAA.

### Dendritic cells

DCs were found in all inflammatory infiltrating and contacting lymphocytes in specimens from patients with AAA, suggesting that they are closely related to AAA (100). NET recruits plasmacytoid dendritic cells, induces IFNs and elastase activation, and promotes AAA development in neutrophil-derived dipeptidyl peptidase I -deficient mice (101). Conditional depletion of CD11C^+^ cells in the ApoE^(−/−)^ mice model of AAA induced by infusion of AngII reduced the maximum diameter of AAA, suggesting that DCs contribute to the development of AAA (102). Previous studies have shown that oral administration of anti-CD3 antibodies or active vitamin D3 reduces AS in mice by recruiting tolerogenic DCs to gut-associated lymphoid tissues (103, 104).

**Figure 2:**
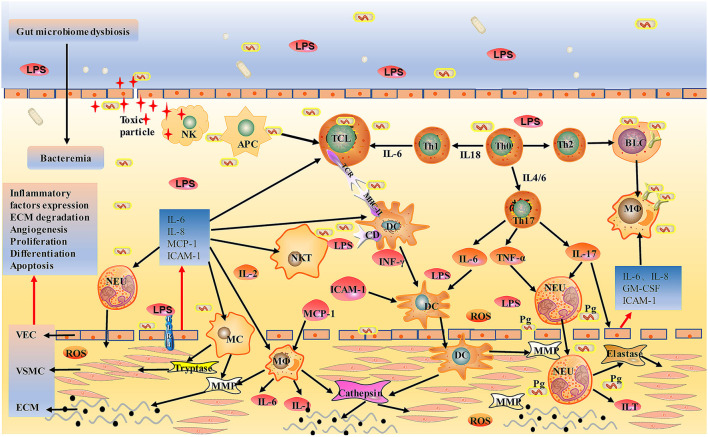
Schematic of gut bacterial infection mediated direct injury to aorta. After entering the circulation, gut bacteria can be engulfed by immune cells or directly invade the wall of the aorta. Bacterial LPS induces DC, MC, and MΦ to overexpress TLR4 and activates them into endothelial cells, releasing chymase, tryptase, and tissue-damaging hist proteases, destroying the aortic wall. Injured vascular endothelial cells produce IL-6, IL-8, MCP-1, ICAM-1, activate circulating TC, BC, DC, MC, NK, NKT, and MΦ, and promote their local adhesion and penetration, leading to metastasis to lesions. LPS also induces Th0 differentiation into Th1 and Th2, further activating effector T cells and plasma cells that directly kill bacteria. NK cells are activated by cytokines, bind to infected vascular endothelial cells, release toxic particles, and induce apoptosis. *Pg* participates in the onset of AAA through neutrophil activation, and neutrophil activation is associated with the formation of NET in ILT. AAA is eventually led by mechanisms, such as extracellular matrix degradation, abnormalities of angiogenesis, proliferation, differentiation, apoptosis, and atherosclerosis. MCP-1, monocyte chemoattractant protein-1; ICAM-1, Intercellular adhesion molecule-1; *Pg, Porphyrinum gingivalis*; NET, neutrophil extracellular traps; ILT, intraluminal thrombosis.

**Figure 3:**
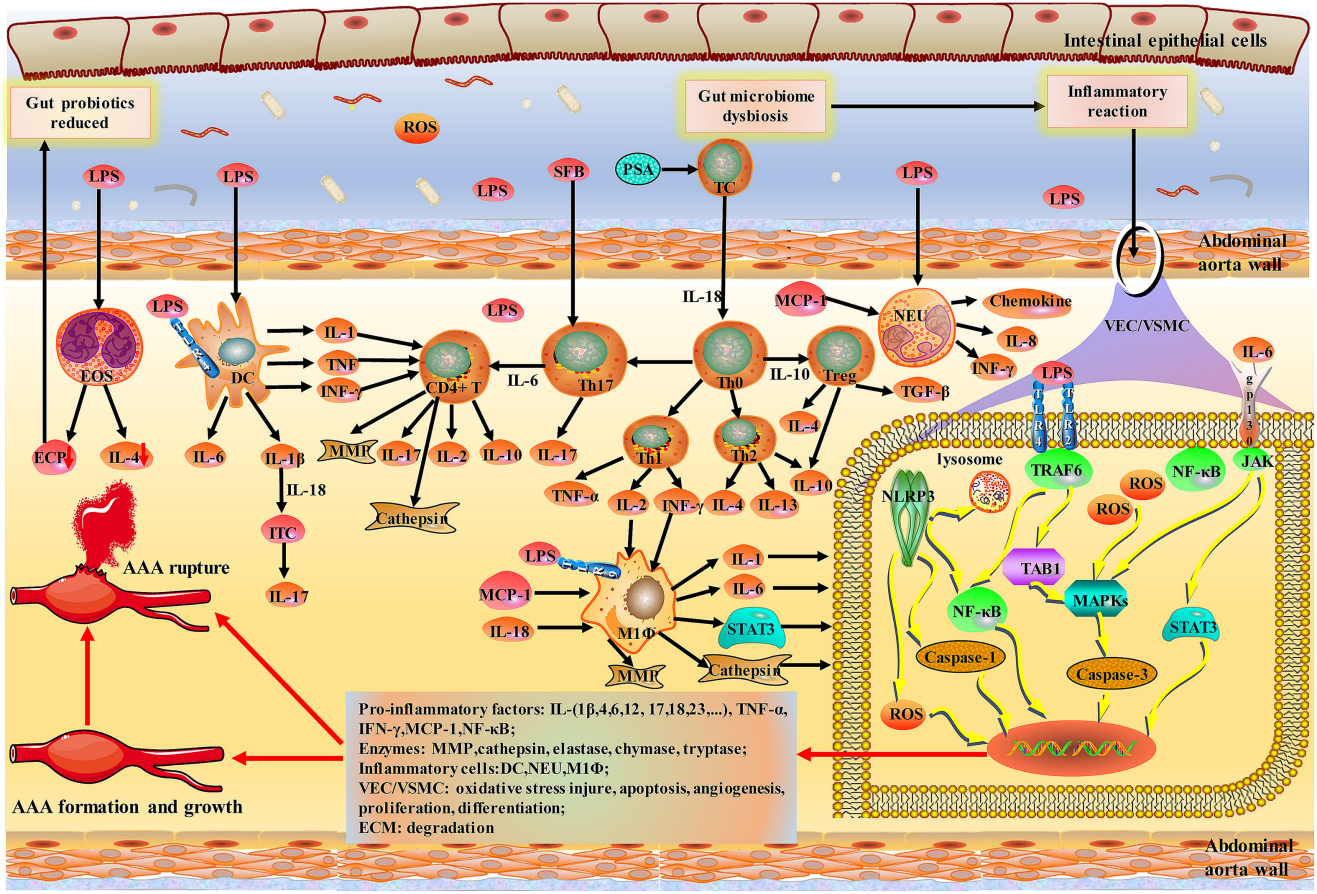
Schematic of indirect damage to AAA caused by inflammatory response to the gut-aortic axis. *Bacteroides fragilis* PSA has been shown to impact Th1/Th2 ratio. NLRP3 inflammasomes, members of the NOD-like receptor (NLRs) family, are widely present in vascular endothelial cells and various immune cells. Activation of TLR and NLRP3 inflammasomes during initial immunity can exacerbate vascular wall damage through caspase-mediated apoptosis. MCs, MΦ, and NEU are activated by LPS and then release a variety of pro-inflammatory factors. EOS upregulation of IL4 regulates the polarization of MΦ and monocytes and blocks NF-κB activation in aortic inflammation and vascular wall cells. Notably, IL-6 can chemotact monocytes/macrophages and activate STAT3 and MCP-1 to promote the expansion of the abdominal aorta. Various inflammatory factors and enzymes lead to the formation, growth and rupture of AAA. PSA, polysaccharide A; NLR, NOD-like receptor; TLRs, toll-like receptors.

Together, the gut microbiota mediated directly or indirectly damage to the abdominal aorta mainly through various inflammatory cells; the diagram for the mechanism is shown in Figures 2, 3.

## Effect of metabolites of the gut microbiome on the pathogenesis of AAA

### Short-chain fatty acids

Gut dysbiosis leads to a decrease in SCFAs and exacerbated inflammation and pulmonary HTN (105). Normal SCFA-producing bacteria include *Bacteroides, Prevotella, Iprevo, Ricinobacter butyricum, Eubacterium, Stinrobacter*, and *Clostridium* IV (92). *Bacteroides* mainly produce acetate and propionate, while *Firmicutes* mainly produce butyrate (106) and propionate, promoting extrathoracic Treg differentiation (107). Butyrate can also cause gut macrophages and DCs to downregulate LPS-induced pro-inflammatory cytokine production (i.e., NO, IL-6, and IL-12), further supporting its role as an anti-inflammatory metabolite, forming the anti-inflammatory effect of SCFAs (108). Besides, SCFAs also increase the levels of glucagon-like peptide-1 to improve insulin resistance, and these factors are closely related to the pathogenesis of AAA (109).

### Trimethylamine N-oxide

The bacteria that produce TMA/TMAO are mainly *Clostridium, Vibrio desulfuricus, Enterobacter*, and *Escherichia coli*. A previous study found that the proportions of *Klebsiella, Pseudomonas, Roche, Proctor, Clostridium, Staphylococcus, Streptococcus, Citrobacter*, and *Coriolis* were significantly increased in patients with pulmonary arterial HTN, which was negatively related to the production of TMA/TMAO (110). TMAO has been shown to be closely related to CVD, and increased plasma TMAO can promote the formation of AS, leading to platelet hyperreactivity and foam cell formation in the aortic root, and can be related to future major adverse cardiovascular events (111). TMAO also promotes the proliferation and migration of VSMCs by upregulating the secretion of inflammatory factors by macrophages (112). Additionally, 3,3-dimethyl-1-butanol can reduce the production of TMAO by inhibiting distinct microbial TMA lyases and alleviating vascular remodeling (112, 113), which will help to inhibit the progression of AAA.

### Indole

Indole is produced by various symbiotic gram-positive and gram-negative bacteria, such as *Escherichia coli, Prevotella*, and *Bacteroides* (110). Gut microbiome imbalance can lead to abnormal tryptophan metabolism, which increases the level of 3-hydroxy-o-aminobenzoic acid through the transcription factor NF-κB, which upregulates MMP2, resulting in the occurrence of AAA (114). Indoleamine 2-3 dioxygenase 1 (IDO) knockout can prevent VSMC apoptosis in AngII-treated LDLr^(−/−)^ mice fed an HFD, indicating that IDO plays a harmful role in the formation of AAA and may be an important target (115). Tryptophan metabolites have both high inflammatory and anti-inflammatory effects, which can cause the development of AS and aneurysms; thus, targeting the tryptophan metabolic pathway will likely assist with AAA treatment (116).

### GABA

GABA can exert its protective effect against vascular endothelial cells damaged. Oral GABA can inhibit the activity and proliferation of APCs and T cells to reduce the inflammatory response (117). GABA deficiency in humans causes not only mental disorders but also abnormal regulation of blood pressure, inflammatory reactions, and AAA (118, 119). Indeed, Afroz et al. (120) showed that the offspring of patients with HSDs had reduced levels of *Lactobacillus*, which may hinder the expression of GABA receptors in the male offspring of HSD parents, leading to neurodevelopmental disorders. Moreover, Topiramate, a GABA receptor agonist, can attenuate experimental AAA progression by promoting macrophage preservation and conversion of M1 to M2 macrophage phenotypes (118).

**Figure 4:**
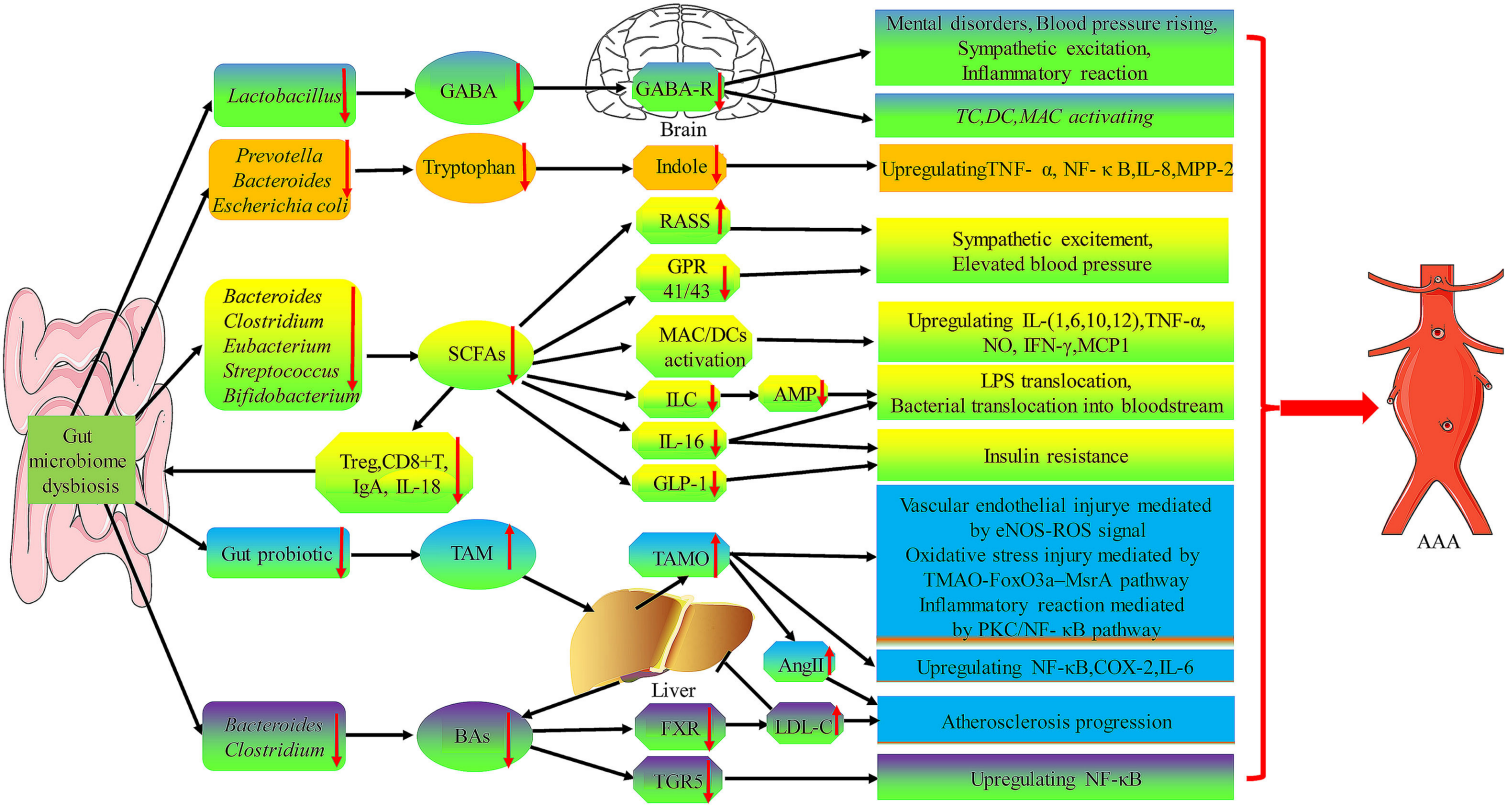
The role of gut microbiota metabolites in the pathogenesis of AAA. Gut microbiome dysbiosis cause the decrease of GABA, Tryptophan, and SCFA produced by certain specific flora, while the increase of TAM and BAs production, which will lead to TC, DC, MAC activating and upregulation of many inflammatory factors (i.e., TNF-α, NF-κ B, IL-1, IL-6, IL-8,IL-12, and MPP-2) to promote AAA formation and progression. GABA, γ-aminobutyric acid; AMP, antimicrobial peptides; GPR, G protein-coupled receptor; ILC, innate lymphocytes; RAAS, renin-angiotensin-aldosterone system; FXR, farnesoid X receptor; TGR5, G-protein-coupled bile acid receptor-1; TMA, Trimethylamine.

Briefly, gut bacteria and metabolites participate in endotoxemia, intestinal permeability change, insulin resistance, hormone environment, gene expression regulating adipogenesis, bile acid interaction, and inflammatory reaction, which play an important role in the pathogenesis of AAA (Figure 4).

## Therapeutic targets mediated *via* the bacterial gut–aortic axis

### Gut probiotics

*Lactobacillus reuteri* can decrease the serum levels of triglycerides, LDL, and HHcy, while *Parabacteroides goldsteinii* reduces the weight of HFD-fed mice, reduces inflammation, and improves insulin resistance (75, 121). Moreover, oral administration of *Lactobacillus brevis* OW38 to aging mice strengthens gut barrier junctions, reduces circulating LPS levels and pro-inflammatory cytokine expression, and inhibits NF-κB activation (122). *Lactobacillus plantarum* HAC01 improves metabolic disorder in HFD-induced diabetic mice through the regulation of the gut microbiota (61). *Shewanella marinintestina* MCCC1 A01703 isolated from the gut tract of marine animals can produce Eicosapentaenoic acid and prevents AAA formation and development by inhibiting the Tak-1-JNK-MMP9 pathway (123). Itaconic acid, which is decomposed by the filamentous fungus *Aspergillus terreus* (124), inhibits AAA formation by inhibiting vascular inflammation, and treatment with increased itaconic acid may help to prevent AAA formation (125). Recently, a new generation probiotic, *A. muciniphila*, was found to be significantly reduced in AAA mice, and it was found to improve AS and repair the damaged gut barrier in ApoE^(−/−)^ mice AS model (126). *A. muciniphila* has also prominence in weight loss, lipid-lowering, blood glucose control, insulin resistance reduction, and anti-inflammatory effects (127). Moreover, genistein, an active isoflavone, alleviates insulin resistance and the inflammatory response by regulating the abundance of genera *Bacteroides, Prevotella, Helicobacter*, and *Ruminococcus* in HFD- and streptozotocin-induced T2DM mice (128).

Prebiotics and synbiotics have prominent advantages in improving gut microbiome imbalance and inflammatory state and have become safe options for next-generation therapeutics of chronic diseases and improvement of human health in recent years (129) and may provide prospects for AAA treatment. ECP stimulates the growth of SCFA-producing bacteria, increases the level of SCFAs in the gut, and for the first time, demonstrated that ECP is a novel prebiotic for health promotion and management of dissonance-related diseases (92). Synbiotics are mixtures of prebiotics and probiotics that significantly reduced the risk of cardiovascular and metabolic syndromes and insulin resistance in elderly patients (130), which not only increase the number of gut probiotics, *Bifidobacterial* and *Lactobacillus*, but also reduce the proportion of the *Coliform* group (131). Antibacterial peptides, mainly originating from bacitracin, *gramicidin S*, and *polyxin E*, can prevent LPS from binding to TLR4 and triggering inflammation and are expected to be used to treat AAA by inhibiting the vascular inflammatory response (132).

### Antibiotics

Doxycycline, a broad MMP inhibitor, can prevent aneurysm growth, and life-threatening aneurysm rupture, and reduce the need for expensive invasive therapy in patients with small AAA (133). An interesting report has shown that 2-weeks of doxycycline treatment before aneurysm repair surgery improved the proteolytic balance in AAA (134). DAV13, a powerful antibiotic adsorbent, decreased the free moxifloxacin fecal concentrations by 99% but largely preserved the richness and composition of the gut microbiota (70). A previous clinical trial has proposed that the administration of roxithromycin could limit the growth of AAA (135). Depletion of the gut microbiota was achieved *via* an oral antibiotic cocktail of vancomycin, metronidazole, ampicillin, and neomycin, to decrease macrophage infiltration and mRNA levels of inflammatory cytokines, which significantly reduced the incidence of aneurysms (136).

### Immune modulators

Rapamycin is a commonly used immunosuppressant in the clinic. After binding with FK binding protein, rapamycin can inhibit mTOR function. Rapamycin changes not only the host gene expression profile but also the gut subgenome in mice (137). Moreover, an HFD can upregulate the active expression of aortic macrophages, MCP-1, and MMPs, which can be inhibited by rapamycin (41). Resveratrol is a specific inhibitor of the mechanistic target of rapamycin complex 1, which can reduce *Lactococcus, Clostridium* XI, *Oscillibacter*, and *Hydrogenoanaerobacterium*, and can improve the glucose intolerance and insulin resistance of HFD-fed mice (138). Moreover, Sirtuin1 can effectively block NF-κB and MCP-1 from initiating an inflammatory response in VSMCs, representing an important target for preventing the formation of AAA (139). Besides, selectively blocking IL-6 signal transduction with sgp130 can improve the survival rate of AAA mice (140). Cilostazol could reduce macrophage accumulation, MMPs activation, and inflammatory gene expression in the aortic media, which may be a promising new therapeutic option for inhibiting the occurrence and growth of AAA (141). Montelukast, a cysteinyl leukotriene receptor 1 antagonist, also induces M2 macrophage polarization and suppresses gene expression of MMP2, MMP9, and IL-1β, which inhibits murine AAA formation (43). Calcitriol also significantly decreases macrophage infiltration, neovessel formation, and MMP2, MMP9, and vascular endothelial growth factor expression in the suprarenal aortic walls; thus, oral calcitriol can reduce dissecting AAA formation (142). Although the inhibition of MC activity may be a target of AAA therapy, the MC stabilizing drug of pemirolast is ineffective in limiting the progression of AAA (143). A recently discovered novel chemokine, FAM3D, was also found to be significantly upregulated in human AAA tissues, and the application of a FAM3D neutralizing antibody was shown to significantly inhibit the formation of AAA and the infiltration of neutrophils (144).

### Nitric oxide

Nitric oxide (NO), which is produced by nitrate-reducing bacteria including *Veillonella, Actinomyces, Haemophilus*, and *Neisseria* (145), is involved in neurotransmission, nerve transmission, vasodilation, and gastrointestinal motility (146), and it has physiological functions in the vascular endothelium *via* NO synthases. Additionally, macrophages are stimulated by inflammatory cytokines, such as TNF-α, IL-1, and IFN-γ, which can produce NO (147). It has been found that local infection with or dissemination of bacteria causes impairment of NO bioavailability and low circulatory levels of NO, leading to endothelial-dependent vascular dysfunction and the formation of atherosclerotic plaques (148). Evidence has shown that restoring the oral flora and NO activity by utilizing probiotics may be beneficial in treating HTN (145). Therefore, how to increase NO activity is considered a potential therapeutic strategy to treat AAA.

### Antihyperlipidemic drugs

Cholesterol-lowering drugs of the statin family are potent inhibitors of HMGCoA reductase and reduce serum CRP and IL-6 levels significantly (149). Although statins are effective in treating AS by lowering LDL, current cardiovascular strategies aimed at lowering LDL may not prevent AAAs (150). High-density lipoprotein, injected into *P. gingivalis*-induced AAA rats, led to a significant reduction in AAA diameter and neutrophil activation and effectively prevented AAA progression (151). Additionally, ezetimibe reduced proteolysis and inflammation through MMP9 and IL-6 in the aortic wall to inhibit the progression of AAA (152). *Corydalis bungeana*, a Chinese herbal medicine, not only has anti-obesity and lipid-lowering effects but also positively regulates the gut S microbiome (40).

### Fecal microbiota transplantation

Clinically, FMT is mainly used to treat bacterial infections (153) and other non-infectious diseases, such as obesity, diabetes, metabolic syndrome, cancer, and Parkinson's disease, most of which are AAA risk factors (154). Therefore, FMT may also play a role in treating AAA. Recently, capsule-based FMT has been proven to be a clinically effective method to restore the composition of gut microbiota, which provides a simple route of administration and flexibility for clinicians and patients (155).

In summary, the bacterial–gut–aortic axis provides a potential measure for targeting AAA. The full list of interventions is listed in Supplementary Table 2.

## Conclusion

We summarized the evidence and risk factors of AAA related to the gut microbiome. The pathological mechanism is mainly based on the direct injury of the gut microbiome to the abdominal aortic wall and the indirect effect of the inflammatory reactions mediated by the gut microbiome imbalance on AAA. The outlined studies prove the importance of the gut microbiome in the pathogenesis of AAA and reveal new targets that can be used to treat AAA. Studying the role of pathophysiological factors related to the intestinal microbiome in the occurrence and development of AAA will provide a new direction for the pathogenesis, biomarkers, and drug R&D of AAA.

## Author contributions

JG, XL, and TL conceived and designed the study. XL and WJ wrote the manuscript. XQ, SZ, and KS collected the literature. All authors read and approved the final manuscript.

## Funding

This work was supported by grants from the Hainan Province Science and Technology special fund (ZDYF2020214 to JG and ZDYF2020122 to WJ), the National Natural Science Foundation of China (82170440 and U220A20270 to JG, 81860075 to TL, and 82260083 to WJ), the National Science Fund for Distinguished Young Scholars (JBGS202104 to JG), Hainan Provincial Nature Foundation (821QN0986 to XL), and the Cardiovascular Disease Research Science Innovation Group of Hainan Medical University.

## Conflict of interest

The authors declare that the research was conducted in the absence of any commercial or financial relationships that could be construed as a potential conflict of interest.

## Publisher's note

All claims expressed in this article are solely those of the authors and do not necessarily represent those of their affiliated organizations, or those of the publisher, the editors and the reviewers. Any product that may be evaluated in this article, or claim that may be made by its manufacturer, is not guaranteed or endorsed by the publisher.
